# ePathOptimize: A Combinatorial Approach for Transcriptional Balancing of Metabolic Pathways

**DOI:** 10.1038/srep11301

**Published:** 2015-06-11

**Authors:** J. Andrew Jones, Victoria R. Vernacchio, Daniel M. Lachance, Matthew Lebovich, Li Fu, Abhijit N. Shirke, Victor L. Schultz, Brady Cress, Robert J. Linhardt, Mattheos A. G. Koffas

**Affiliations:** 1 Department of Chemical and Biological Engineering, Rensselaer Polytechnic Institute, Troy NY 12180, USA; 2 Department of Biological Sciences, Rensselaer Polytechnic Institute, Troy NY 12180, USA; 3 Department of Chemistry, Rensselaer Polytechnic Institute, Troy NY 12180, USA

## Abstract

The ability to fine tune gene expression has created the field of metabolic pathway optimization and balancing where a variety of factors affecting flux balance are carefully modulated to improve product titers, yields, and productivity. Using a library of isopropyl β-D-1-thiogalactopyranoside (IPTG)-inducible mutant T7 promoters of varied strength a combinatorial method was developed for transcriptional balancing of the violacein pathway. Violacein biosynthesis involves a complex five-gene pathway that is an excellent model for exploratory metabolic engineering efforts into pathway regulation and control due to many colorful intermediates and side products allowing for easy analysis and strain comparison. Upon screening approximately 4% of the total initial library, several high-titer mutants were discovered that resulted in up to a 63-fold improvement over the control strain. With further fermentation optimization, titers were improved to 1829 ± 46 mg/L; a 2.6-fold improvement in titer and a 30-fold improvement in productivity from previous literature reports.

Over the past two decades, there has been an enormous push for the microbial production of many fuels, commodity chemicals, and natural products that are currently being environmentally sourced. Efficient microbial production can provide stability for diminishing natural sources and facilitate the use of renewable energy sources such as lignocellulose^1^. Traditional metabolic engineering efforts coupled with synthetic biology tools have allowed the expression of active metabolic pathways from a wide variety of organisms, demonstrating the potential production of thousands of compounds ranging from traditional pharmaceuticals^2^ to advanced biofuels^3^. These efforts have further been improved upon using pathway balancing and optimization strategies to achieve industrially feasible titers, yields, and productivities for a wide range of products^4^,^5^. Although there have been several excellent examples of pathway optimization, there are still many systems with production at non-industrially feasible levels that are in need of optimization to boost titers, yields, and productivities^6^,^7^,^8^.

The challenge of pathway optimization can be addressed by any combination of five predominant methods: DNA^5^,^9^,^10^, RNA^11^, protein^12^, and post-translational^13^ level optimization as well as applications of real-time dynamic balancing^14^. Each method has its own advantages and disadvantages, and therefore the technique(s) selected should be tailored for the specific application. A review discussing each of these methods in detail has been recently published^15^.

Traditionally, the bacteriophage T7 promoter has been widely used for protein expression and purification, but commonly avoided in metabolic engineering applications due to its propensity to overburden the cell with extrinsic mRNAs reducing translation of essential intrinsic mRNAs, subsequently reducing cell health^16^,^17^. The strength of the consensus promoter is also attributed to its propensity for leaky expression even under T7-lac control^18^. Here we demonstrate an up-front RNA-level optimization method, harnessing and controlling the strongly inducible T7 promoter for the efficient production of violacein in *E. coli*.

The violacein biosynthetic pathway was chosen as a model pathway for the application of this technology, due to its pathway complexity as well as it being a proven bioactive compound demonstrating a wide range of antibiotic^19^, antifungal^20^, as well as *in vitro*^21^ and *in vivo*^22^ anticancer properties. Several recent excellent reviews have been written summarizing the potential applications of violacein in the pharmaceuticals, cosmetics, textiles, food, and pesticide industries^23^,^24^,^25^,^26^,^27^.

The violacein pathway (Fig. 1) consists of five pathway genes culminating in the production of violacein (and deoxyviolacein, due to promiscuity of the VioC enzyme,) and several additional intermediate and side products proceeding through spontaneous or unknown aerobic reactions. This pathway mimics the complexity encountered in many other microbial pathways and therefore represents an excellent model pathway for the demonstration of our developed technology. The ability to transcriptionally control the violacein pathway has been successfully demonstrated in yeast using constitutive promoters^28^.

Here we demonstrate a method for the creation and characterization of a functional T7 promoter library, as well as a cloning strategy, utilizing ePathBrick architecture^29^, for quick and easy combinatorial library incorporation into pathway construction. Using traditional restriction/ligation cloning, the five-gene violacein pathway was assembled with randomized promoters in under a week. Upon screening less than four percent of possible combinations, several high-titer mutants were discovered that showed up to 63-fold improvement over the control strain that contains all strong consensus promoter sequences controlling the expression of the violacein pathway. With further fermentation optimization, the highest reported violacein titer to date, 1.83 ± 0.05 g/L was achieved representing a 260% improvement over previous benchmarks in *E. coli*^30^.

## Results

### Development and characterization of a T7 promoter library

**Figure 2:**
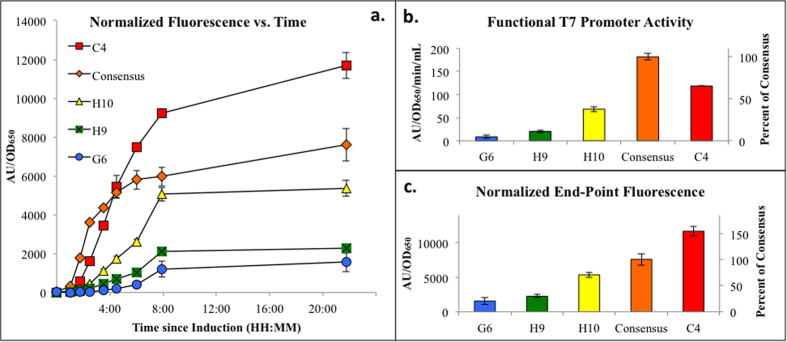
Analysis of mutant T7 promoter strength. (**A**) Increase in fluorescence normalized by OD_650_ versus time for the 5-member promoter library. (**B**) Functional promoter activity calculated as the slope of the linear region of subset A. (**C**) Functional promoter strength assessed by final end-point fluorescence normalized by cell density (OD_650_). Error bars represent ±1 standard deviation of biological triplicate.

The first step in the present study was to select the sequences that will represent the functional promoter library to be used for later pathway optimization trials. The mutant library was constructed using site-directed mutagenesis. The library of mutant plasmids was then screened for end-point and time-based mCherry expression. The functional activity of each mutant was then assessed using two metrics: absolute expression and expression change with respect to time. A five member functional library was then selected to represent the large dynamic range of mutants and to highlight any interesting mutants observed in the library. Analysis of the results (Fig. 2) highlights the interesting reversal of the consensus sequence and mutant C4 between the two analysis methods. Although the consensus sequence displays the steepest slope through their respective linear regions, the mutant C4 contains a longer linear region consequently resulting in higher endpoint fluorescence.

### Initial screening of violacein production demonstrates the power of the method

**Figure 3:**
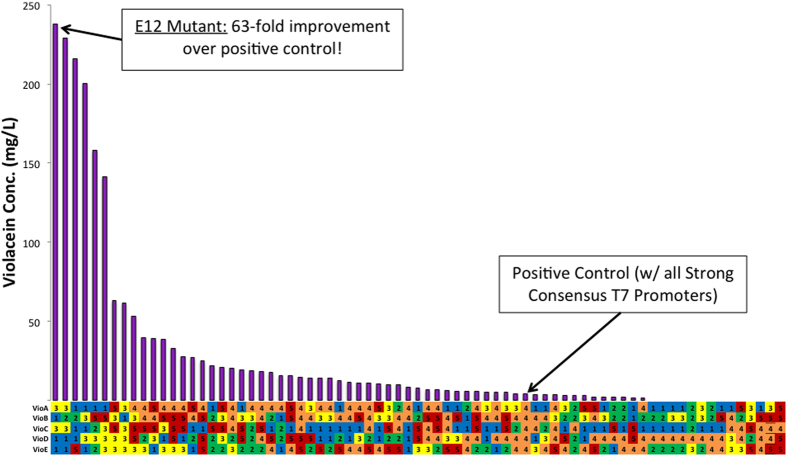
Initial screening of violacein production from randomized promoter library. Several high-titer mutants were discovered through screening less than 4% of the total library size (107/3125). Sequence analysis of the library is displayed below the horizontal axis. The 5 members of the promoter library were assigned numbers and colors from low to high as follows: G6: 1, Blue; H9: 2, Green; H10: 3, Yellow; Consensus: 4, Orange; C4: 5, Red. Initial screening was preformed using *E. coli* BL21star^TM^(DE3) in LB Media at 30 °C. Cultures were induced after 4.5 h of growth with 1 mM IPTG. Violacein was analyzed by HPLC after methanol extraction.

BL21star^TM^(DE3) colonies, each containing a different randomized combination of promoters for each of the five violacein pathway genes (*vioABCDE*), were evaluated for violacein production. Initial screening of 107 colonies (3.4% of the theoretical library) revealed large improvements in violacein production when compared to the control strain containing the consensus sequence (Fig. 3). The top producing strain, E12, produced 238 mg/L of violacein as compared to the control strain that produced only 3.8 mg/L. This represents a 63-fold improvement under initial screening conditions (LB media, 30 °C). Each of the library members was then sequenced to determine the strength of the promoter used for each gene in the pathway. The sequencing results are displayed under the horizontal axis (Fig. 3). Initial screening demonstrated approximately one-third of the libraries members show no violacein production. After sequence analysis, it was determined that the large majority (70%) of these non-producing strains were cloning malfunctions, lacking 1 or more of the pathway genes. These members were removed from data analysis (Fig. 3).

### Optimization of media and fermentation temperature for improved violacein production

**Figure 4:**
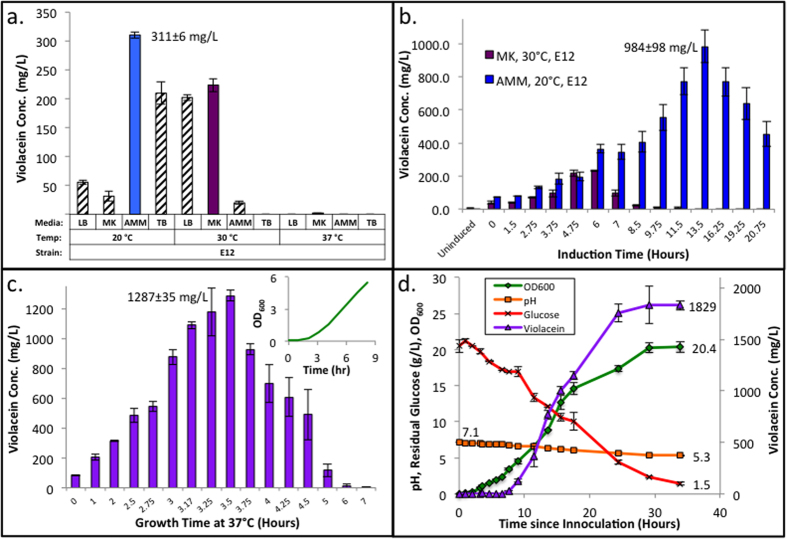
Optimization of fermentation conditions for peak violacein production from strain E12. (**A**) Media and temperature optimization show dependent effects for both parameters on violacein production. The two top conditions (AMM, 20 °C) and (MK, 30 °C) were used for further optimization. Error bars represent ± 1 standard deviation of biological triplicate. (**B**) Induction point optimization of the best conditions as determined in subset A. Time on the x-axis indicates the time between inoculation and induction. Data indicates that fermentation in AMM at 20 °C results in significantly improved peak production over MK media at 30 °C. Error bars represent ±1 standard deviation of biological triplicate. (**C**) Induction point optimization under improved conditions. Time on the x-axis represents the time the culture was grown at 37 °C. The cultures were then transferred to 20 °C for 1 h prior induction with 1 mM IPTG. Significant improvement in violacein titers were shown by cell growth at 37 °C followed by protein induction and fermentation at 20 °C. Error bars represent ±1 standard deviation of biological duplicate. (**D**) Demonstration of scale-up potential. Peak production conditions from subset C were used to show violacein production in a 500 mL culture contained in a 2-L baffled shake flask. Significant improvements in titer were obtained presumably due to the differences in growth dynamics and dissolved oxygen levels in baffled shake flask. The larger fermentation volume also allowed for transient analysis of fermentation pH, glucose consumption, absorbance at 600 nm, and violacein production rate. Error bars represent ±1 standard deviation of biological duplicate.

After discovery of several top producing violacein strains, we set to optimize the fermentation conditions for violacein production for the top producing strain, E12 (238.1 mg/L, Fig. 3). We began by testing several media (LB, TB, AMM, and MK) and fermentation temperatures (37, 30 and 20 °C), motivated by previous literature reports^30^ and personal observations. Our findings (Fig. 4a) demonstrate strong influence of both factors on production. It is shown that LB and MK media show elevated production at 30 °C, while AMM and TB media show improved production at 20 °C. Overall, the highest production of 310.6 mg/L was obtained in AMM at 20 °C, representing a 30% increase in titer over the initial screening parameters.

### Induction point optimization further improves violacein titers

The top two conditions from the previous study (AMM, 20 °C; MK, 30 °C) were then further improved by varying the time between inoculation and induction of protein expression with IPTG. Results (Fig. 4b) show peak production for the MK, 30 °C case near the values previously reported (Fig. 4a), while it was clear that the AMM, 20 °C cultures were induced too early (Fig. 4a) with peak values after 13.5 h of growth. We then hypothesized that we could decrease fermentation time while maintaining high titers by applying different temperatures to the growth and production phases. This was accomplished by first growing cells at 37 °C then cooling them to 20 °C for induction^31^. Using this fermentation strategy, while optimizing induction time showed marked improvements over previous methods (Fig. 4c, 1287 mg/L, 31% improvement). Peak production was achieved by allowing cell growth at 37 °C for 3.5 h (OD_600_ ≈ 1.2) followed by acclimation at 20 °C for 1 h prior to induction with 1 mM IPTG (OD_600_ ≈ 1.5).

### Half-liter shake flask fermentation demonstrates scalability of production

We then attempted to show scalability of production by scaling-up from 25 mL culture (in 125 mL shake flask) to 500 mL culture (in a 2 L baffled shake flask). In this experiment, we used the best conditions from previous studies with additional transient sampling and analysis of pH, glucose, optical density (OD_600_), and violacein production. Analysis revealed further improvements in titer with scale-up resulting in a final violacein concentration of 1829 ± 46 mg/L with average productivity of 103 mg/L/h during the linear production period (7.6–24.4 h). This represents the highest titer and productivity for violacein produced in *E. coli* to date.

### Enrichment of Violacein Library

**Figure 5:**
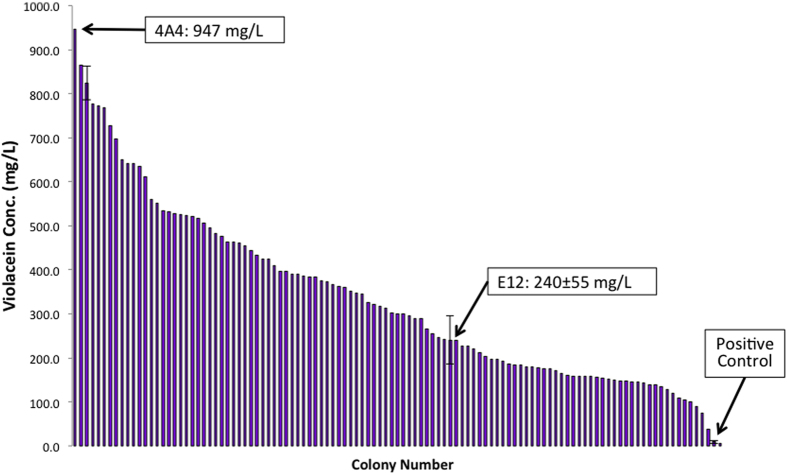
Screening of enriched violacein production library. Substantial improvements are seen when compared to the initial screen (Fig. 3). Mutant E12 (top mutant from initial screen), positive control, and the top mutant 4A4 are highlighted. All screening was performed using *E. coli* BL21star^TM^(DE3) in LB Media at 30 °C. Cultures were induced after 4.5 h of growth with 1 mM IPTG. Violacein was analyzed by HPLC after methanol extraction. Error bars represent ±1 standard deviation of biological duplicate.

Analysis of the sequencing results coupled with the initial violacein production screen revealed that the high-titer mutants contained an increased percentage of reduced strength promoters (G6, H9, and H10). In an attempt to improve the initial library, a second enriched library was constructed that only included the three reduced strength promoters using the ePathOptimize system. Upon screening 200 mutants (82% of theoretical library size), the results showed substantial improvements in both the proportion of high-titer mutants as well as the titer of the top-producing mutants (Fig. 5). The best mutant, 4A4, produced a final titer of 947 mg/L under initial screening conditions.

## Discussion

This work demonstrates the importance of not only developing superior production strains from a genetic standpoint but also highlights the importance of tailored fermentation conditions for optimized microbial production. The current method for developing optimized and efficient production strains commonly involves the selection of a library of different biological parts, combining these pieces with various plasmid backbones, and subsequently conducting individual screens of each configuration until a top producer is found. This cumbersome method often results in optimized systems with multiple plasmids, restricted cell growth, and little room for improvement or modification.

Here we present a method for pathway optimization, in which an entire metabolic pathway is built into a single plasmid in less than one week, allowing for rapid library construction and screening. Detailed timeline for randomized-promoter plasmid construction can be found in supplementary materials. This process utilizes a library of five characterized T7 mutant promoters with reduced strength, further simplifying the process by having the entire pathway contained on a single plasmid backbone. This ePathBrick-based technology can theoretically be employed with up to three plasmids concurrently allowing for modularized optimization of entire metabolic systems.

Upon characterization of the T7 promoter library, it was observed that the protein expression from the consensus T7 sequence displayed a short linear region of very rapid protein accumulation followed by reduction in expression rate and a leveling-off of total fluorescence. This is contrasted with the expression of the C4 mutant T7 promoter, which displayed an extended linear region of protein production before quickly leveling off. We predict that this interesting behavior of the consensus promoter is linked to its ability to quickly produce high levels of recombinant protein and its less than desirable performance in metabolic engineering applications. Guided by previous literature findings^16^, we hypothesize that this rapid protein (and, presumably, mRNA) production creates unnecessary stress on the cells by redirecting many necessary cellular resources, towards extrinsic protein (and mRNA) production. This outcome is preferred when the desired product is recombinant protein, but it is undesirable when cellular processes are needed for precursor, co-factor, or biomass production. This discovery provides further evidence that reduced strength expression could in fact lead to more production through reduction of metabolic burden. The presented method has extensive applicability to a wide variety of pathways. Our lab has demonstrated the use of ePathBrick vectors for the production of flavonoids^29^, fatty acids^5^, and polysaccharides^10^ indicating these vectors are applicable for expression of both endogenous and exogenous genes in *E. coli*. The use of sequence-verified parts reduces the risk of PCR-introduced errors in library construction that are inherent to other methods.

Results (Fig. 3) above highlight the potential that metabolic pathway optimization can have on extrinsic pathways. Violacein was chosen as a model pathway for the proof of principle of this technology, due to its pathway complexity as well as being a potent bioactive compound that demonstrates a wide range of antibiotic, antifungal, and anticancer properties as well as a promising potential for use as a bio-dye for food, cosmetics, and fabric coloring. Through screening of approximately 4% of the total library size, several high titer mutants were discovered, with one producing 63-fold more violacein than the positive control strain. The positive control refers to each gene’s transcription being controlled by the strong T7 consensus promoter. We sequenced the 107 screened mutants, determining the strength of the promoters controlling transcription for each gene in the pathway, to further investigate the cause for the increased violacein production. The goal of this analysis was to develop a hypothesis as to why some mutants had very high violacein production while others had very low production. Initial sequence analysis revealed a strong trend between high titer mutants and weakened promoter sequences. Of the top 8 mutants with the highest titer, 87.5% (35/40) of the promoter sequences were found to be weaker than consensus (H10, H9, or G6). Furthermore, 100% of the remaining low production mutants contained at least one strong promoter sequence (consensus or C4). These results make a compelling case for using reduced strength promoters for metabolic engineering applications involving the expression of heterologous pathways, although many factors such as RBS strength, plasmid copy number, as well as application specific parameters must also be considered before any overarching conclusions can be stated.

We then constructed an enriched library including only the three weakened promoters to further expand upon these findings. This allowed us to screen a larger proportion of the total library while simultaneously enriching the weakened promoter/high-titer region (Fig. 3). Upon screening, we discovered a greater proportion of high-titer mutants and a nearly 4-fold improvement in the top producing strains under initial screening conditions (Fig. 5). It also seems that with increased library size, conclusions could be reached as to the sensitivity of each pathway gene with respect to overall pathway performance. From a metabolic burden perspective, the lowest net promoter strength required to obtain the top production would be preferred. No clear trends were evident in the presented data, indicating that a larger percentage of the total library must be sampled before definite conclusions can be made. This ability to use initial screening data to support rational library design and enrichment at an individual gene level is a major advantage of the ePathOptimize system.

Optimizing fermentation conditions for the peak production of violacein resulted in an additional 7.7-fold improvement over genetic optimization for the E12 strain (Fig. 4). These additive effects for both genetic and fermentation conditions optimization indicate the importance of both for improving product titers, yields, and productivity. For this system, the largest improvements were found during induction point optimization trials (Fig. 4b,c). Significant improvements and declines in production were seen on time scales as small as fifteen-minute differences in induction point. Sensitivity at small time scales such as these is rarely investigated for biological systems. This further complicates the complete optimization of microbial production, but it leaves hope that substantial improvements can be obtained through careful optimization. Under optimal conditions and after scale-up, our top producing strain showed a 2.6-fold improvement in titer and a 30-fold improvement in productivity over the highest reported production to date in *E. coli*^30^.

This work highlights the importance of pathway balancing and fermentation optimization studies for the development of microbes capable of industrially feasible metabolite production. Here we present a case study where, through application of transcriptional balancing and basic fermentation optimization, yields of violacein were improved from 3.8 mg/L to 1.83 g/L. This represents a 480-fold improvement in titer, without external tryptophan supplementation or background strain improvements to improve flux towards tryptophan. The generalized method presented above can be quickly and easily applied to a wide variety of systems to improve yields, titers, and productivity. As microbial production systems become more advanced the need for fine-tuning of expression systems will become greater than ever. The development of dynamic regulation systems^14^ are further expanding the microbial engineering tool box, giving scientists never before seen levels of control over biological systems. Future applications of this technology towards improving the microbial production of biofuels and pharmaceuticals could revolutionize/expedite the transition from the laboratory to the marketplace.

## Methods

### Bacterial Strains, Vectors, and Media

*E. coli* DH5α was used to propagate all plasmids, while the BL21star^TM^(DE3) was used as the host for violacein production. The violacein pathway genes were obtained from *Pseudoalteromonas luteoviolacea B*^32^. The ePathBrick vector, pETM6, was used as the basis for all plasmid construction and pathway expression. Luria Broth (LB) Lennox modification (Sigma), Terrific Broth + 2% Glucose (TB)^33^, MK Media^5^, and Andrew’s Magic Media (AMM)^10^ were used where noted. AMM is rich semi-defined media developed from modified protocols^34^,^35^ (3.5 g/L KH_2_PO_4_, 5.0 g/L K_2_HPO_4_, 3.5 g/L (NH_4_)_2_HPO_4_, 2 g/L casamino acids, 100 mL of 10x MOPS Mix, 1 mL of 1 M MgSO4, 0.1 mL of 1 M CaCl2, 1 mL of 0.5 g/L Thiamine HCL, supplemented with 20 g/L glucose). 10x MOPS Mix consisted of 83.72 g/L MOPS, 7.17 g/L Tricine, 28 mg/L FeSO_4_·7H_2_O, 29.2 g/L NaCl, 5.1 g/L NH_4_Cl, 1.1 g/L MgCl_2_, 0.48 g/L K_2_SO_4_, 0.2 mL Micronutrient Stock. Micronutrient Stock consisted of 0.18 g/L (NH_4_)_6_Mo_7_O_24_, 1.24 g/L H_3_BO_3_, 0.12 g/L CuSO_4_, 0.8 g/L MnCl_2_, 0.14 g/L ZnSO_4_.

### T7 promoter library creation and characterization

The T7 promoter library was constructed by standard site-directed mutagenesis (SDM) protocols using complementary primer pairs with mutagenesis being facilitated by degenerate nucleotides replacing the five base pair T7 RNA polymerase binding strength site^36^. The mutagenesis was completed using pETM6-mCherry (consensus T7 promoter) as template DNA. The PCR product was then directly digested with *Dpn*I to remove template DNA, and transformed into chemically competent DH5α. All colonies from the transformation were collected and plasmid DNA was extracted. No liquid media growth was allowed to protect against growth selection. The plasmid DNA was then transformed into BL21star^TM^(DE3) for screening and characterization.

Individual colonies were inoculated into a 1 mL overnight culture of LB Media with ampicillin at 80 μg/mL, 37 °C. After 14 h of growth, the cultures were inoculated (50:1) into 2 mL of LB media with appropriate antibiotics, allowed to grow at 37 °C for 3-4 h before induction (OD_600_ of 1–1.5) with 1 mM IPTG. Transient and end-point OD_650_ and fluorescence data was collected using the BioTek Synergy 4 plate reader with black-walled 96-well plates (Greiner Bio One, Polystyrene). Path length was corrected such that the A_650_ is equivalent to a 1 cm cuvette. All samples were diluted into the linear range for the instrument. Absorbance was measured at 650 nm such that mCherry expression did not interfere with the readings (Supplementary Fig. 1). mCherry fluorescence was measured at Excitation 588 nm, Emission 618 nm. Ampicillin was supplemented where needed at a final concentration of 80 μg/mL.

A five-member library was then selected to cover a wide range of expression levels. Mutant promoter sequences are given in Table 1. The selected members were then digested with *Avr*II and *Sal*I and ligated into new pETM6 backbone to eliminate the possibility of unintended mutations from the SDM PCR amplification. The mCherry open reading frame was also replaced with another fluorescence reporter, eGFP, to confirm that similar trends are obtained for other proteins (Data not shown).

### Violacein pathway cloning and application of T7 promoter library

All five genes constituting the violacein biosynthetic pathway were acquired through PCR amplification (ACCUZYME 2x mix, Bioline) of genomic DNA from *Pseudoalteromonas luteoviolacea* strain B (ATCC 29581)^32^ with the primers—each possessing an *Nde*I restriction site in the forward primer and an *Xho*I restriction site in the reverse primer—number 1–10 in Supp-Table 2. The ePathBrick destination vector pETM6 and all PCR amplicons were digested with restriction enzymes *Nde*I/*Xho*I (FastDigest, Thermo Scientific) and gel purified (E.Z.N.A. MicroElute Gel Extraction Kit, Omega Bio-tek). Individual digested amplicons were ligated with digested pETM6 backbone to create plasmids 2–6, Supp-Table 1. Constructs were then transformed into chemically competent DH5α for verification and plasmid propagation. Colonies were screened via restriction digest and further verified with Sanger sequencing (GENEWIZ, Inc.) using the sequencing primers 11–15 in Supp-Table 2.

The pathway genes in pETM6 were digested with *Nde*I and *Sal*I and gel extracted to obtain the ORF to be used as insert to apply the promoter library to the violacein pathway. The promoter library plasmids were then pooled in equal molar quantities, digested with *Nde*I and *Sal*I, and gel extracted, as a whole, resulting in backbone with randomized promoter. These two fragments were then ligated and transformed, resulting in a random mix of one of the five promoters of varied strength and the pathway gene. The plate(s) were then scraped using sterilized razor blades after 18 hours of growth at 37 °C and the plasmid DNA extracted (no liquid culture growth) to obtain a pool of all randomized members. An appropriate number of colonies were collected to insure retention of library size, typically 5–10 times theoretical library size. This process was then repeated using ePathBrick monocistronic assembly (Supplementary Fig. 2) until all pathway genes were assembled on a single vector. Occasionally the restriction site *Apa*I was used to replace *Sal*I when the pathway genes either contained internal *Sal*I restriction sites or to optimize the insert:backbone ratio for improved ligation efficiency. Multiple transformations were oftentimes completed to ensure sufficient library sampling and retention. The final plasmid library, pETM6-xxVioA-xxVioB-xxVioE-xxVioC-xxVioD, was transformed into BL21star^TM^(DE3) for screening. The ‘xx’ feature represents the inclusion of a single random mutant T7 promoter from the selected library.

### Initial violacein screening with randomized promoter strength

Single colonies were inoculated into 1 mL of LB media with appropriate antibiotics and grown overnight at 37 °C. After 12–16 h, cultures were inoculated into 2 mL of LB media and allowed to grow at 30 °C for 4.5 h before induction with 1 mM IPTG. The fermentation was then grown for 20 h at 30 °C before violacein was extracted and analyzed. All initial screening was completed in polypropylene 48-well plates (5 mL, VWR).

### Analytical methods

Cells were pelleted (20,000 × *g*, 10 min) and the supernatant was removed and used for glucose analysis. The violacein was then extracted from the pellet by adding twice the original culture volume of pure methanol and boiling in a 95 °C water bath for 5 min or until the pellet appeared completely white. In samples with elevated violacein levels, subsequent extractions were required. The extract was then centrifuged (20,000 × *g*, 10 min) to pellet cell debris and 10 μL of extract was directly injected into the HPLC.

Violacein analysis was carried out using Agilent 1200 series HPLC with diode array detector (DAD) and ZORBAX SB-C18 StableBond analytical column (150 mm × 5 mm, 5 μm) maintained at 30 °C. The mobile phases were acetonitrile (A) and water (B), both containing 0.1% formic acid. The following gradient was used at a flow rate of 1 mL/min: 0 min, 5% A; 1 min, 5% A; 5 min, 45% A; 7 min, 55% A; 9 min, 95% A; 10 min, 5% A; 12 min, 5% A. Violacein (7.95 min) and deoxyviolacein (9.11 min) were analyzed by peak area integration at 565 nm using a standard curve. A typical HPLC chromatogram is shown as Supplementary Fig. 3 demonstrating less than ten percent accumulation of deoxyviolacein and no detection of prodeoxyviolacein (7.4 min) or proviolacein (6.8 min).

Glucose was analyzed by injecting 15 μL the supernatant obtained above into an Aglient 1200 series HPLC equipped with a refractive index detector (RID) held at 40 °C and a ZORBAX carbohydrate analysis column (150 mm × 5 mm, 5 μm) maintained at 35 °C. The mobile phase was 75% acetonitrile/25% Water at a flow rate of 1 mL/min. Glucose was quantified using a standard curve.

### Violacein purification and development of a violacein standard curve

Violacein standards were developed using a crystallization protocol adapted from the literature^37^. Violacein was extracted with LCMS grade methanol (95 °C water bath) from 1 L of pelleted culture (25,000 × *g*, 30 min). The resulting crude extract was evaporated to dryness using a Buchi Rotovapor R-210 under reduced pressure. The solid crude violacein was then dissolved in acetone to saturation and filtered to remove and any remaining solids. The acetone extract was then brought to a boil and combined with an equal volume of boiling water. The mixture was placed at 20 °C overnight to allow for crystallization. The crystals were collected via centrifugation (25,000 × *g*, 45 min) and washed twice with MilliQ water and three times with hexanes before drying under reduced pressure. The resulting crystals were then further purified by preparative HPLC (Shimadzu Prominence Series) using a Waters Nova-Pak C18 column (60 Å, 19 mm × 200 mm, 6 μm) at room temperature. One milliliter of concentrated sample in methanol was injected. The mobile phases were acetonitrile (A) and water (B), both containing 0.1% formic acid. The following gradient was used at a flow rate of 10 mL/min: 0 min, 5% A; 3.5 min, 5% A; 17.5 min, 45% A; 24.5 min, 55% A; 31.5 min, 95% A; 35 min, 5% A; 50 min, 5% A. Violacein (28 min) and deoxyviolacein (31.5 min) were separately collected and appropriate fractions evaporated under reduced pressure. The resulting solids were then weighed and dissolved in LCMS grade methanol for standards. A typical preparative HPLC chromatogram is shown (Supplementary Fig. 4). Sample purity was accessed by HPLC (Supplementary Fig. 5), Thin layer chromatography (TLC) (Supplementary Fig. 6), and H-NMR (Supplementary Fig. 7). HPLC and TLC revealed no visible contamination with violacein fraction (3) demonstrating higher percent violacein than Sigma standard (5). H-NMR demonstrates higher sample purity than Sigma standard.

## Additional Information

**How to cite this article**: Jones, J. A. *et al.* “ePathOptimize: A Combinatorial Approach for Transcriptional Balancing of Metabolic Pathways”. *Sci. Rep.* **5**, 11301; doi: 10.1038/srep11301 (2015).

## Supplementary Material

Supplementary Information

## Figures and Tables

**Figure 1:**
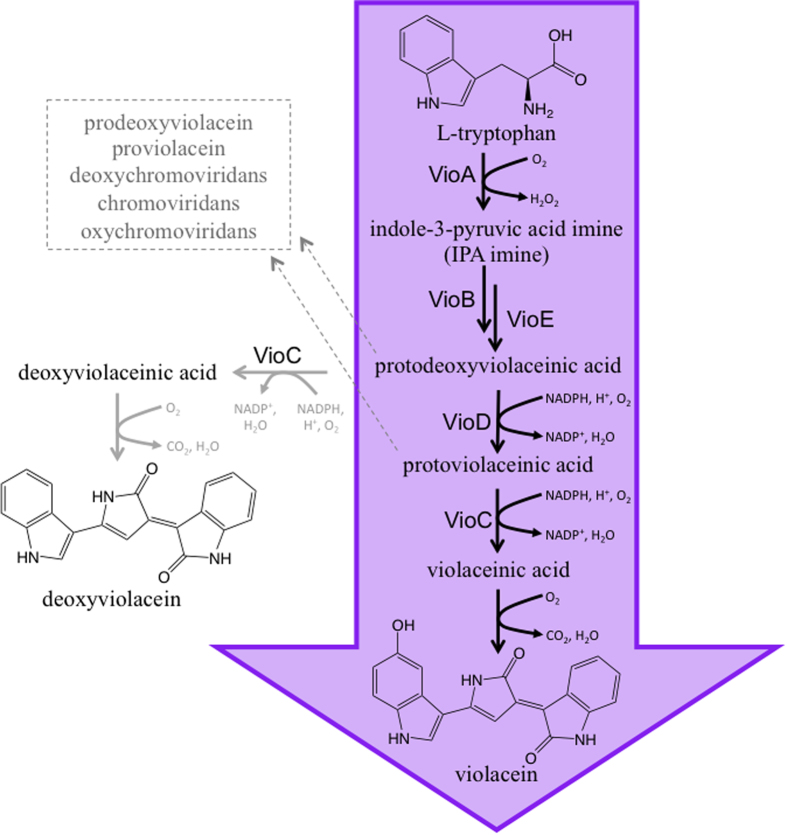
The violacein biosynthetic pathway. Violacein is produced through a 5-step enzymatic process followed by a single non-enzymatic step. Potential side products are shown. The strains constructed in this work typically produced a mixture of violacein (~85–95%) and, the unavoidable side product, deoxyviolacein (~5–15%) with very low to not detectible (Limit of detection: 1 mg/L) production of both other products.

**Table 1:**
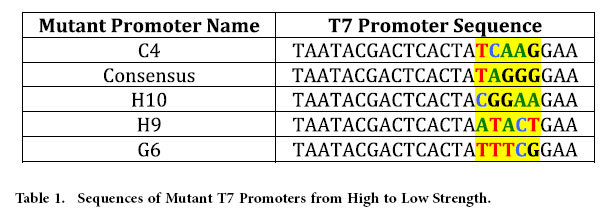
Sequences of Mutant T7 Promoters from High to Low Strength.
