# Supplementary material for: ePathOptimize: A Combinatorial Approach for Transcriptional Balancing of Metabolic Pathways
Source: Sci Rep. 2015 Jun 11;5:11301. doi: 10.1038/srep11301 (PMC4650668; doi:10.1038/srep11301)
Supplement: Supplementary Information [file srep11301-s1.pdf]

Supplementary Figures and Tables

"ePathOptimize: A Combinatorial Approach for Transcriptional Balancing of Metabolic Pathways"

J. Andrew Jones[1], Victoria R. Vernacchio[1], Daniel M. Lachance[2], Matthew Lebovich[1], Li Fu[3], Abhijit N. Shirke[3], Victor L. Schultz[3], Brady Cress[1], Robert J. Linhardt[1,2,3], Mattheos A. G. Koffas[1,2,]*

[1]Department of Chemical and Biological Engineering, Rensselaer Polytechnic Institute, Troy NY 12180, USA
[2]Department of Biological Sciences, Rensselaer Polytechnic Institute, Troy NY 12180, USA
[3]Department of Chemistry, Rensselaer Polytechnic Institute, Troy NY 12180, USA
* Corresponding Author

*Corresponding Author Information
Mattheos A. G. Koffas
Rensselaer Polytechnic Institute
110 8th Street Troy, NY 12180
(518) 276-2220
koffam@rpi.edu

Supplementary Discussion: Cloning Timeline for 5-gene pathway

Day 1: Total Time (7 Hours)
3 Hours - PCR amplification of 5 pathway genes
1 Hour - Digestion of destination vector and PCR products with Thermo FastDigest restriction enzymes NdeI and XhoI
1 Hour - Gel Electrophoresis and Gel Extraction of appropriate fragments
2 Hours - Rapid DNA Ligation (Thermo) and Transformation into *E. coli* DH5α

Day 2: Total Time (9 Hours)
8 Hours - Pick colonies in to LB media and allow 7 Hours for growth
1 Hour - Miniprep resulting cultures and send for same day sequencing

Day 3: Total Time (2 Hours)
2 Hours - Analyze sequencing results and start overnight cultures for positive clones

Day 4: Total Time (5 Hours)
1 Hour - Miniprep overnight cultures of positive clones
1 Hour - Digest plasmids containing each pathway gene and pooled promoter library plasmids with NdeI and SalI
1 Hour - Gel electrophoresis and gel extraction of appropriate fragments.
2 Hours - Rapid DNA Ligation (Thermo) of each gene with randomized backbone and transformation into *E. coli* DH5α

Day 5: Total Time (5 Hours)
1 Hour - Scrap plates and miniprep culture
1 Hours - Digest randomized plasmid containing genes 1 and 3 with NheI and SalI, and randomized plasmid containing genes 2 and 4 with AvrII and SalI
1 Hour - Gel electrophoresis and gel extraction of appropriate fragments.
2 Hours - Rapid DNA Ligation (Thermo) of genes 1-2 and 3-4 and transformation into *E. coli* DH5α

Day 6: Total Time (5 Hours)
1 Hour - Scrap plates and miniprep culture
1 Hours - Digest randomized plasmid containing genes 3-4 with NheI and SalI, and randomized plasmid containing gene 5 with AvrII and SalI
1 Hour - Gel electrophoresis and gel extraction of appropriate fragments.
2 Hours - Rapid DNA Ligation (Thermo) of genes 3-4 with gene 5 and transformation into *E. coli* DH5α

Day 7: Total Time (5 Hours)
1 Hour - Scrap plates and miniprep culture
1 Hours - Digest randomized plasmid containing genes 1-2 with NheI and SalI, and randomized plasmid containing gene 3-4-5 with AvrII and SalI
1 Hour - Gel electrophoresis and gel extraction of appropriate fragments.
2 Hours - Rapid DNA Ligation (Thermo) of genes 1-2 with genes 3-4-5 and transformation into *E. coli* DH5α

Day 8: Total Time (1 Hour)
1 Hour – Scrap plates and miniprep culture – This is the 5-gene pathway on a single plasmid with randomized promoters.

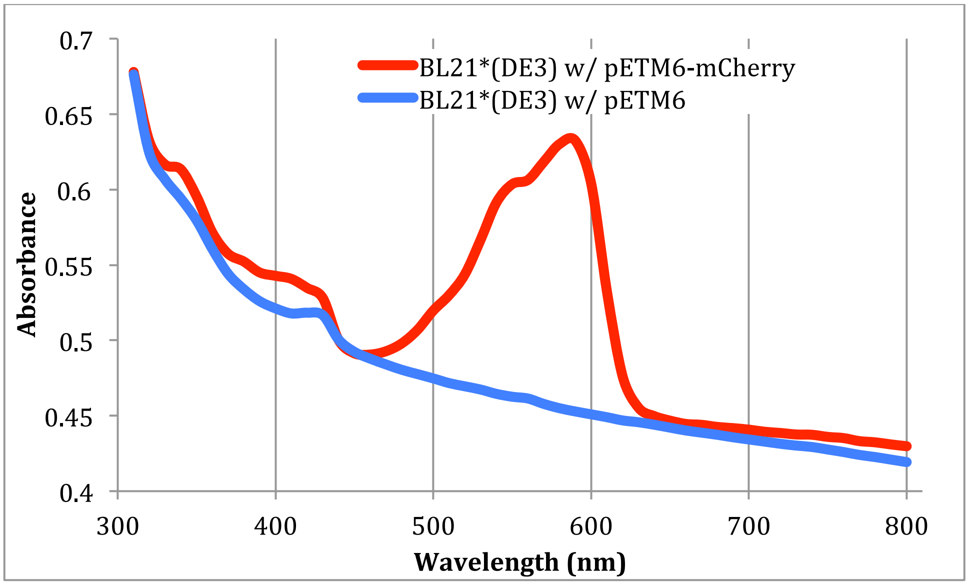

Supplementary Figure 1. Absorbance spectrum of *E. coli* BL21star^{TM}(DE3) expressing the fluorescent reporter mCherry. Note that the control strain (not expressing mCherry) has a smooth profile while the cells with mCherry present have considerable interference from 450 nm to 630 nm. Optical density measurements for fluorescence studies were taken at 650 nm to minimize interference from mCherry.

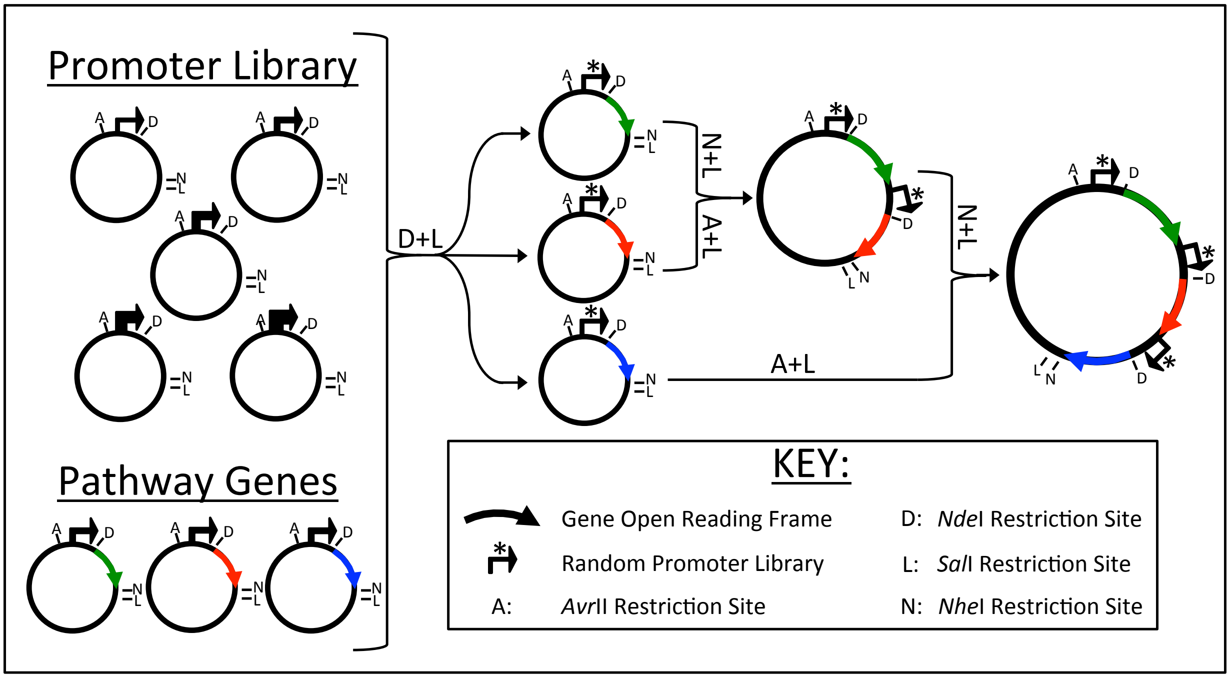

Supplementary Figure 2. Cloning scheme for incorporation of a T7 promoter library into ePathBrick architecture. Utilization of isocaudomer restriction enzyme pairs enables the rapid construction of entire pathways onto a single plasmid. Digestion of pooled, equal molar concentrations of plasmid containing characterized promoters allows for ultimate flexibility in library design.

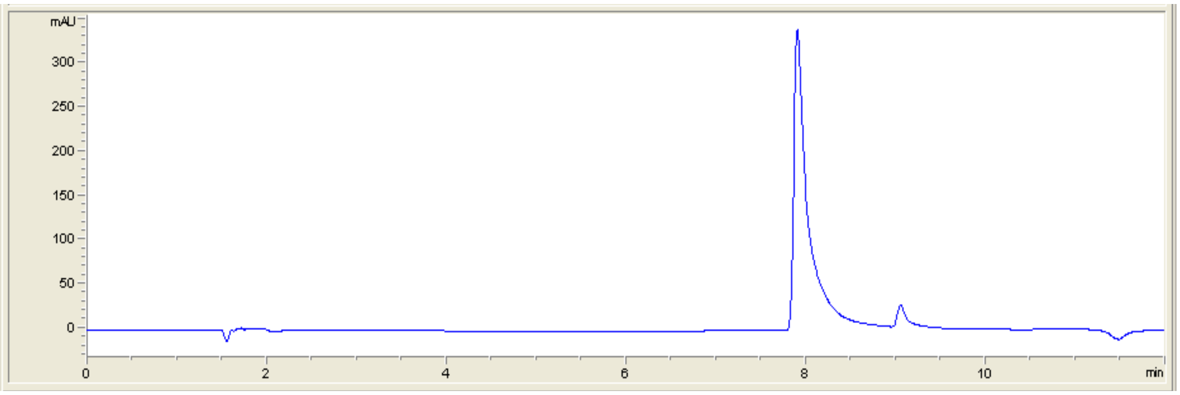

Supplementary Figure 3. Typical HPLC chromatogram used for violacein quantification at 565 nm. Violacein eluted at 7.95 min and deoxyviolacein eluted at 9.11 min.

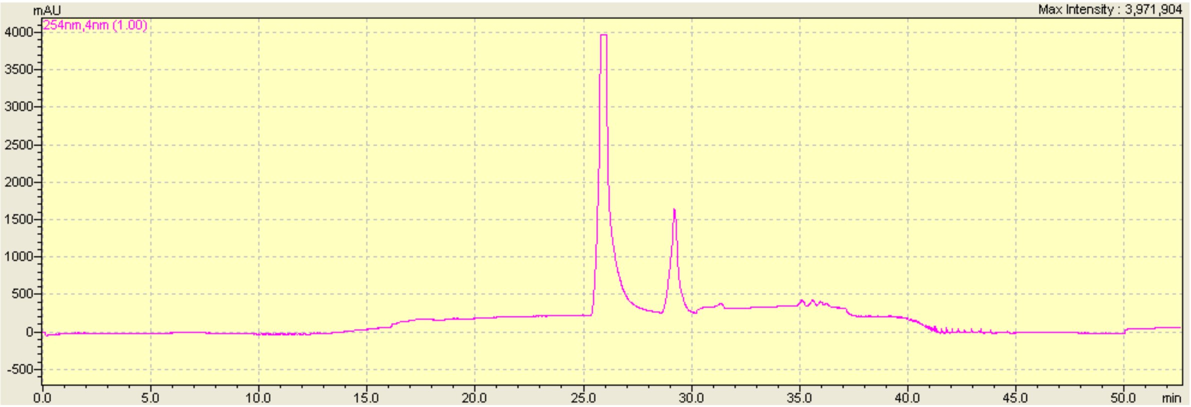

<u>Supplementary Figure 4.</u> <u>Typical preparative HPLC chromatogram for purification of violacein standard.</u> Violacein eluted at 26.5 min and deoxyviolacein eluted at 29.5 min.

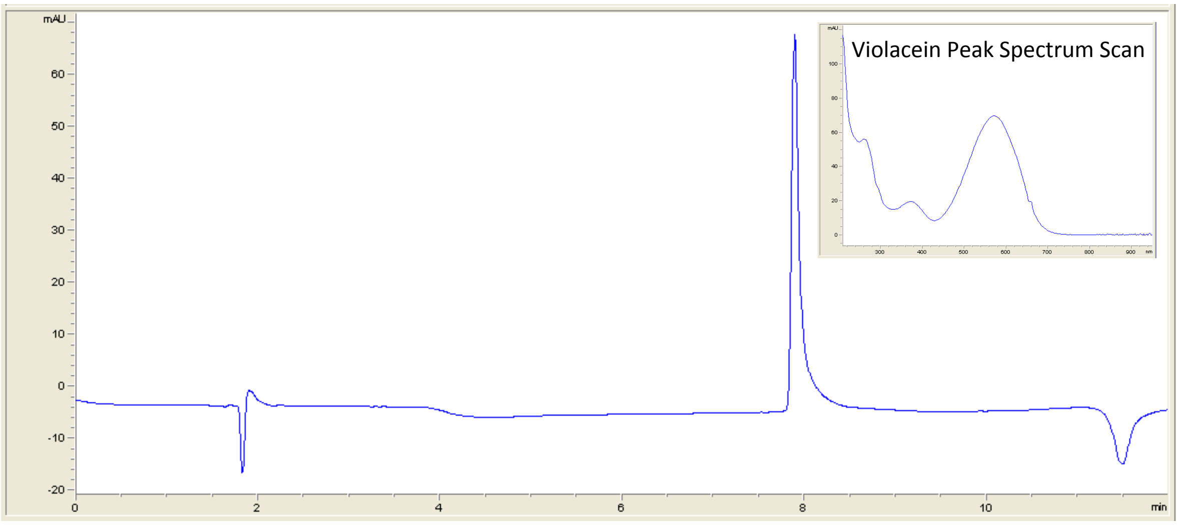

<u>Supplementary Figure 5.</u> <u>Analytical HPLC chromatogram for violacein fraction from preparative HPLC run</u>. Only violacein peak is present at 7.9 min. Insert shows UV spectrum scan of violacein peak. This is consistent with literature reports.

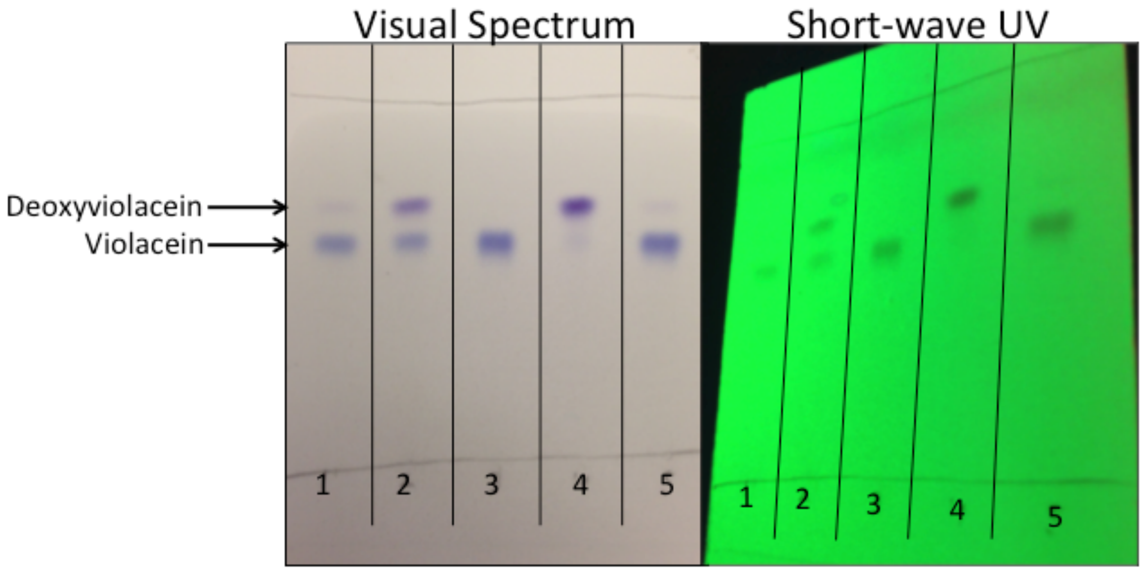

Supplementary Figure 6. Thin layer chromatography (TLC) for violacein samples at various levels of purification. (1) Crude violacein just after methanol extraction. (2) After crystallization and hexane extraction. (3) Violacein fractions after preparative HPLC. (4) Deoxyviolacein fractions after preparative HPLC. (5) Sigma standard (Violacein >85%). Sample 3 shows higher percentage violacein than Sigma standard (5).

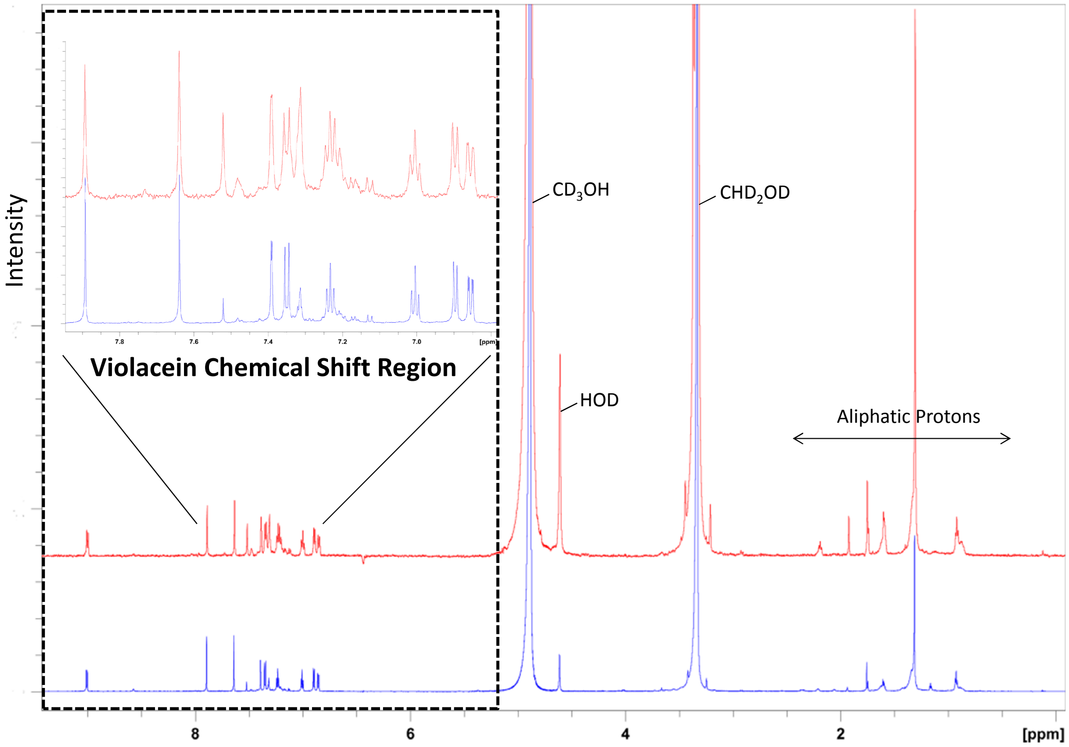

Supplementary Figure 7. Proton NMR (600 MHz) spectra of violacein prepared from *E. coli* BL21*(DE3) expressing the violacein pathway from plasmid (Blue), and violacein prepared from *Janthinobacterium lividum* (Red, Sigma). Initial structurally informative aromatic proton signals are observed between 6-9 ppm are shown expanded (insert). Signals from NMR solvent (CD_3OH) and trace water 4.0-5.5 ppm are labeled. Aliphatic signals 0.5 – 2.5 ppm observed, probably correspond to residual fatty acids/lipids.

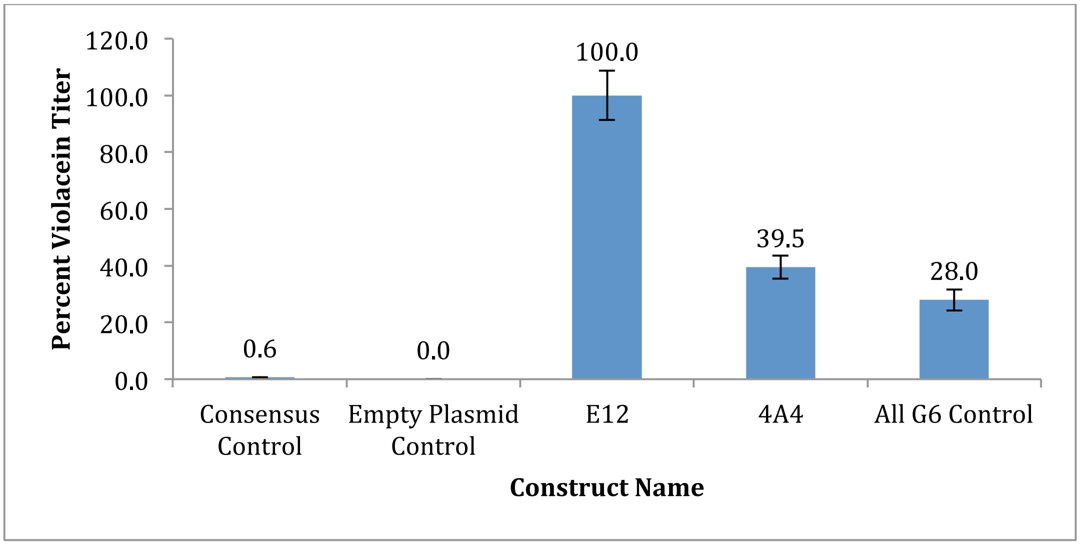

<u>Supplementary Figure 8.</u> Comparison of top producing strains under conditions optimized for mutant E12. Although construct 4A4 produced significantly higher violacein titers under the initial screening conditions (See Figure 5), when compared to construct E12 under conditions optimized for construct E12, its final titer was only 40% that of E12. This supports the case that both genetic and fermentation optimization are necessary for maximum production. The 'All G6 Control' represents each gene of the violacein pathway expressed from our weakest strength promoter. This shows that the trivial (all low) solution is also not sufficient for optimal production. Experiments were conducted in 48-well plate.

Supplementary Table 1. List of strains and plasmids used in this study.

Number	Strain or vector	Relevant properties	Reference
S1	*Escherichia coli* DH5α	F^-, φ80d lacZΔM15, Δ(lacZYA-argF)U169, recA1, endA1, hsdR17(rk^-, mk^+), phoA, supE44λ^-, thi^{-1}, gyrA96, relA1	Novagen
S2	*E.coli* BL21 Star™ (DE3)	F^- *ompT gal dcm rne131 lon hsdS*$_B$ ($r_B^- m_B^-$) λ(DE3)	Invitrogen
S3	*Pseudoalteromonas luteoviolacea*	No modifications: ATCC 29581	(Cress et al., 2013)
1	pETM6	ePathBrick expression vector, ColE1 ori, AmpR	(Xu et al., 2012)
2	pETM6-VioA	#1 containing *vioA* from *P. luteoviolacea*	This Study.
3	pETM6-VioB	#1 containing *vioB* from *P. luteoviolacea*	This Study.
4	pETM6-VioC	#1 containing *vioC* from *P. luteoviolacea*	This Study.
5	pETM6-VioD	#1 containing *vioD* from *P. luteoviolacea*	This Study.
6	pETM6-VioE	#1 containing *vioE* from *P. luteoviolacea*	This Study.
7	pETM6-VioAB	#1 containing *vioA-vioB* in Monocistronic configuration	This Study.
8	pETM6-VioCD	#1 containing *vioC-vioD* in Monocistronic configuration	This Study.
9	pETM6-VioABE	#1 containing *vioA-vioB-vioE* in Monocistronic configuration	This Study.
10	pETM6-VioABECD	#1 containing *vioA-vioB-vioE-vioC-vioD* in monocistronic configuration	This Study.
11	pETM6-mCherry	#1 containing mCherry fluorescent reporter	(Xu et al., 2012)
12	pETM6-C4-mCherry	#11 Modified with mutant 'C4' T7 promoter sequence	This Study.
13	pETM6-G6-mCherry	#11 Modified with mutant 'G6' T7 promoter sequence	This Study.
14	pETM6-H9-mCherry	#11 Modified with mutant 'H9' T7 promoter sequence	This Study.
15	pETM6-H10-mCherry	#11 Modified with mutant 'H10' T7 promoter sequence	This Study.
16	pETM6-eGFP	#1 containing eGFP fluorescent reporter	(Xu et al., 2012)

Supplementary Table 2. List of primers used in this study.

Number	Primer ID	Primer Sequence (5'-->3')
1	vioA_NdeI_F	GCGCCATATGTTAAATTTAGAGCATTCAG
2	vioA_XhoI_R	CGTACTCGAGTCAAAGGTATACTACTTCTTTCAC
3	vioB_NdeI_F	GCACATATGAGTGTTTTAGATTTTCCTCG
4	vioB_XhoI_R	CTCTCGAGCTAACCTTCCTTTGAAAG
5	vioC_NdeI_F	GCGCATATGAGTAAAATAATTATTGTTGGTGGTG
6	vioC_XhoI_R	CGCTCGAGTTAATTCATTCTCCCTATTTTGTAC
7	vioD_NdeI_F	GCACATATGAACATTTTAGTGATCGGG
8	vioD_XhoI_R	GATCTCGAGTTAACGTTGCAGCGC
9	vioE_NdeI_F	GCGCATATGGAATTACGTAAAGTAGATAGAGTTCC
10	vioE_XhoI_R	CGCTCGAGTCAATTCCTATGAGAGAGAC
11	vioB_mid1_seq_F	GCAAGGATTTCTTATTGAATCAATTGGGCTTGC
12	vioB_mid2_seq_F	CCAAACCTAATCGAGTTGCAAAAAAGCAAGC
13	vioB_mid3_seq_F	GCATTAAAAGACTCTGTCGACCTAGAGTTGTCG
14	T7_FWD	TAATACGACTCACTATAGGG
15	T7Term_REV	GCTAGTTATTGCTCAGCGG
16	SDM_PT7_Random_FWD	CCCGCGAAATTAATACGACTCACTANNNNNGAATTGTGAGCGGATAACAATTCCC
17	SDM_PT7_Random_REV	GGGAATTGTTATCCGCTCACAATTCNNNNNTAGTGAGTCGTATTAATTTCGCGGG
18	Seq_Pro_FWD_VioA	CGCTCTCCCTTATGCGACTCC
19	Seq_Pro_FWD_VioB	GGGGCATCTTAGCCGGCAAG
20	Seq_Pro_FWD_VioC	GCAGCGGCAAAAGCGTTAAACC
21	Seq_Pro_FWD_VioD	CCGAACGTTCACGCCTCTGTAAG
22	Seq_Pro_FWD_VioE	CGTCTATGGCGTATAGCGAGCTTC
